# Obstructive jaundice as a rare complication of multiple pancreaticoduodenal artery aneurysms due to median arcuate ligament syndrome: a case report and review of the literature

**DOI:** 10.1186/s13256-023-04114-6

**Published:** 2023-09-10

**Authors:** Javad Jalili, Reza Javadrashid, Dara Alvandfar, Masih Falahatian, Ali Jafarizadeh, Samin Alihosseini, Seyedeh Elnaz Hashemizadeh

**Affiliations:** 1 Medical Radiation Sciences Research Group, Tabriz University of Medical Sciences, Tabriz, Iran; 2 Department of Radiology, Emam Reza Hospital, Tabriz University of Medical Sciences, Tabriz, Iran; 3 Department of General Surgery, Emam Reza Hospital, Faculty of Medicine, Tabriz University of Medical Sciences, Tabriz, Iran; 4 Student Research Committee, Tabriz University of Medical Sciences, Tabriz, Iran; 5 Department of Surgical and Clinical Pathology, Emam Reza Hospital, Tabriz University of Medical Sciences, Tabriz, Iran

**Keywords:** Jaundice, Superior mesenteric artery, Pancreaticoduodenal artery aneurysm, Median arcuate ligament syndrome, Common bile duct, Computed tomography angiography, Case report

## Abstract

**Background:**

Obstructive jaundice has various causes, and one of the rarest is pancreaticoduodenal artery aneurysm (PDAA), which is often associated with celiac axis stenosis caused by median arcuate ligament syndrome (MALS).

**Case presentation:**

The patient was a 77-year-old Azeri woman who presented with progressive jaundice, vague abdominal pain, and abdominal distension from 6 months ago. The intra- and extrahepatic bile ducts were dilated, the liver's margin was slightly irregular, and the echogenicity of the liver was mildly heterogeneous in the initial ultrasound exam. A huge cystic mass with peripheral calcification and compressive effect on the common bile duct (CBD) was also seen near the pancreatic head, which was connected to the superior mesenteric artery (SMA) and had internal turbulent blood flow on color Doppler ultrasound. According to the computed tomography angiography (CTA) findings, the huge mass of the pancreatic head was diagnosed as a true aneurysm of the pancreaticoduodenal artery caused by MALS. Two similar smaller aneurysms were also present at the huge aneurysm's superior margin. Due to impending rupture signs in the huge aneurysm, the severe compression effect of this aneurysm on CBD, and the patient's family will surgery was chosen for the patient to resect the aneurysms, but unfortunately, the patient died on the first day after the operation due to hemorrhagic shock.

**Conclusion:**

In unexpected obstructive jaundice due to a mass with vascular origin in the head of the pancreas, PDAA should be considered, and celiac trunk should be evaluated because the main reason for PDAA is celiac trunk stenosis or occlusion by atherosclerosis or MALS. The treatment method chosen (including transarterial embolization, open surgery, or combined method) depends on the patient's clinical status and radiological findings, but transarterial embolization would be safer and should be used as a first-line method.

## Introduction

The fusiform aneurysm is a focal dilatation of an artery at least 1.5 times its predicted diameter, while a saccular aneurysm is a berrylike outpouching. Both of these conditions are classified as true aneurysms and contain all three layers of a vessel wall. Pseudoaneurysm doesn't have all of these layers and is a consequence of intimal disruption, which leads to abnormal to-and-fro blood flow outside the vessel's lumen [1]. Due to their non-specific symptoms, visceral artery aneurysms (VAAs) are rarely discovered, although their mortality rate is 25–75% [2]. Among the visceral arteries, the pancreaticoduodenal artery (PDA) is less involved [3], and about 2% of VAAs belong to pancreaticoduodenal artery aneurysms (PDAA) [4].

Aneurysms or pseudoaneurysms of PDA are usually caused by celiac artery occlusion or stenosis, pancreatitis, bacterial infection, and iatrogenic trauma from surgery or vascular manipulation [5]. In most cases, PDAA is caused by stenosis or occlusion of the common hepatic or celiac artery due to MALS (median arcuate ligament syndrome) or atherosclerosis [6]. 68% of MALS patients had PDAA, indicating a close link between these conditions [7]. In fact, due to celiac artery stenosis, the arterial pressure gradient in the pancreaticoduodenal arteries reverses, and retrograde blood flow from the superior mesenteric artery (SMA) enters the PDA to supply the organs normally nourished by the celiac trunk [5, 8]. This phenomenon causes hemodynamic stress on these vessels, making them prone to aneurysm formation [9].

PDAAs are usually asymptomatic and are not diagnosed until they rupture. However, due to the increased use of diagnostic modalities today, the rate of accidental diagnosis and reporting of asymptomatic PDAAs has increased [10]. Although, in scarce situations, mass-effect symptoms such as biliary obstruction [11], abdominal pain, abdominal distention, and spontaneous retroperitoneal hemorrhage [12] can be seen. When PDAA is ruptured, the mortality rate reaches 50%, even in asymptomatic cases [13]. So therapeutic interventions are recommended regardless of the size of the aneurysm [14].

In this study, we present an extremely rare case of multiple PDAAs caused by MALS in a 77-year-old woman who presented with obstructive jaundice. Following that, we discuss the imaging findings of this patient with a pathological correlation, as well as a review of the literature on the characteristics of PDAA in the setting of MALS and its treatment approaches.

## Case presentation

The patient was a 77 years old Azeri woman who presented with progressive jaundice, vague abdominal pain, and abdominal distention from 6 months ago. For the past month, the patient has been experiencing multiple episodes of severe consecutive coughing. Outpatient evaluations, including chest computed tomography (CT) scans and Spirometry, did not show any abnormalities. The patient denied a history of smoking, asthma, other chronic pulmonary diseases, recent respiratory tract infections, or taking new medication. Therefore, we started empiric treatment to relieve her coughs, which included omeprazole 40 mg once daily, inhaled budesonide 400 mcg per day, inhaled salbutamol 400 mcg per day, Brompheniramine 6 mg twice daily, and dextromethorphan 20 mg daily. In addition, the patient has had diabetes and hypertension for around 30 years. These conditions are treated with Metformin 500 mg twice daily and Lozartan-H 12.5 mg twice daily, respectively.

Laboratory data showed significantly elevated levels of direct bilirubin and alkaline phosphatase suggestive of cholestasis. Aspartate aminotransferase (AST) and alanine aminotransferase (ALT) levels were also mildly increased. Complete blood count (CBC) was normal, and erythrocyte sedimentation rate (ESR) and c-reactive protein (CRP) levels were not increased. CA 19-9 was in the normal range. In Appendix 1 we present an overview of important laboratory results.

**Fig. 1:**
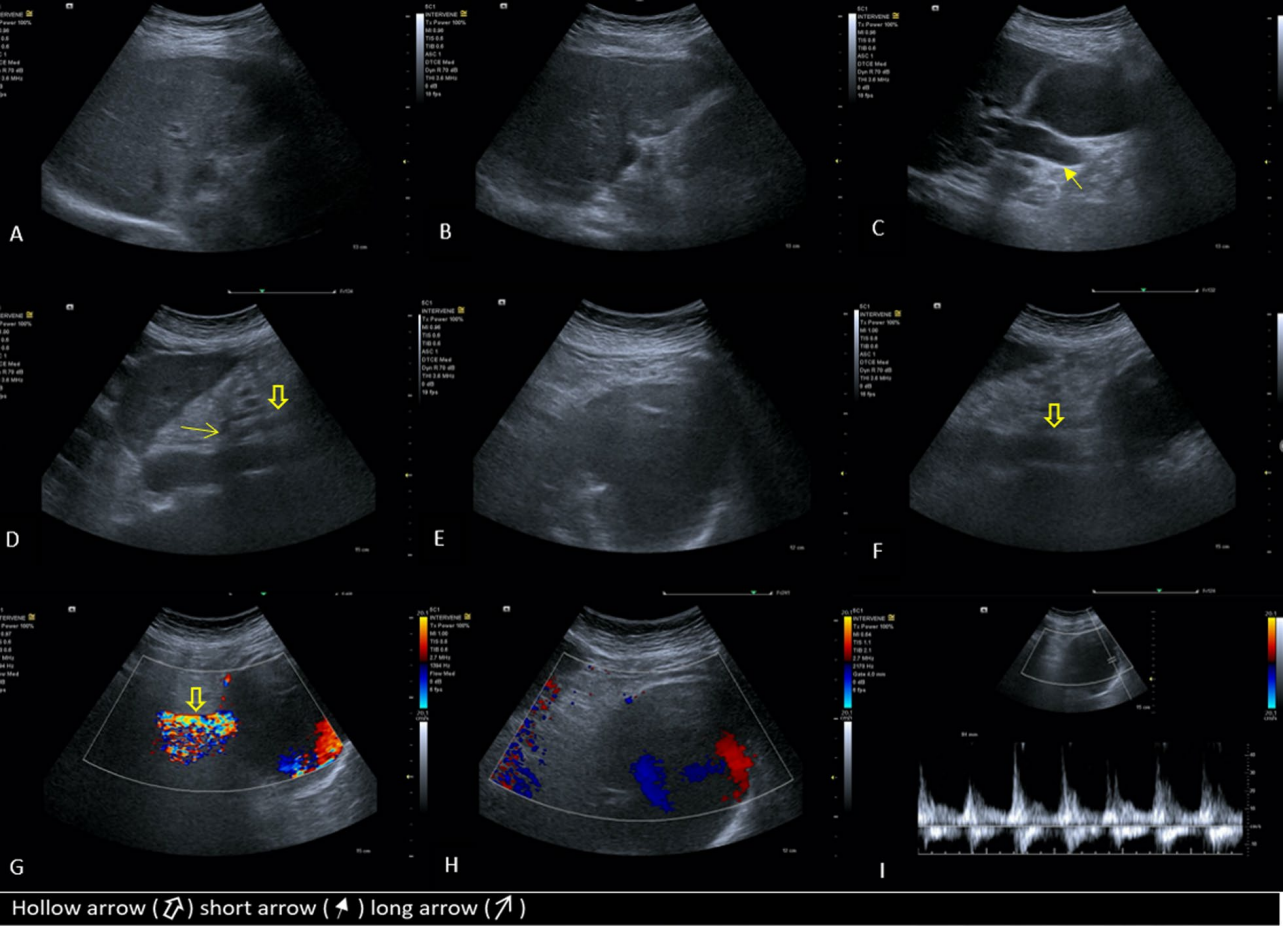
Surface of the liver is irregular and its echogenicity is mildly coarse and heterogeneous. Mild ascites is seen around the liver (**A**, **B**). Common bile duct and intrahepatic bile duct dilation (**C**). Sagittal scan of aorta shows stenosis of proximal of celiac trunk (**D**). The huge aneurysm with “yin and yang” sign on color Doppler ultrasound, and turbulent arterial blood flow on Spectral Doppler (**E**, **H**, **I**). The aneurysm is superiorly connected to superior mesenteric artery. The superior mesenteric artery continues its course on the anterior surface of the aneurysm to supply the small bowel (**F**, **G**). Hollow arrows shows superior mesenteric artery, short arrow shows dilated common bile duct, and long arrow shows celiac trunk

With suspicion of a mass causing obstructive jaundice, ultrasonography was performed. Ultrasonography showed a large cystic mass with peripheral calcification in the head of the pancreas leading to moderate dilation of extra and intrahepatic bile ducts. In the Doppler ultrasound exam, the mass had turbulent blood flow with yin and yang sign and was connected to the superior mesenteric artery, suggesting aneurysmal dilation or pseudoaneurysm of this artery or its branches. Also, the echogenicity of the liver was mildly heterogeneous, and its margin was not completely smooth, in favor of chronic parenchymal liver disease (Fig. 1).

**Fig. 2:**
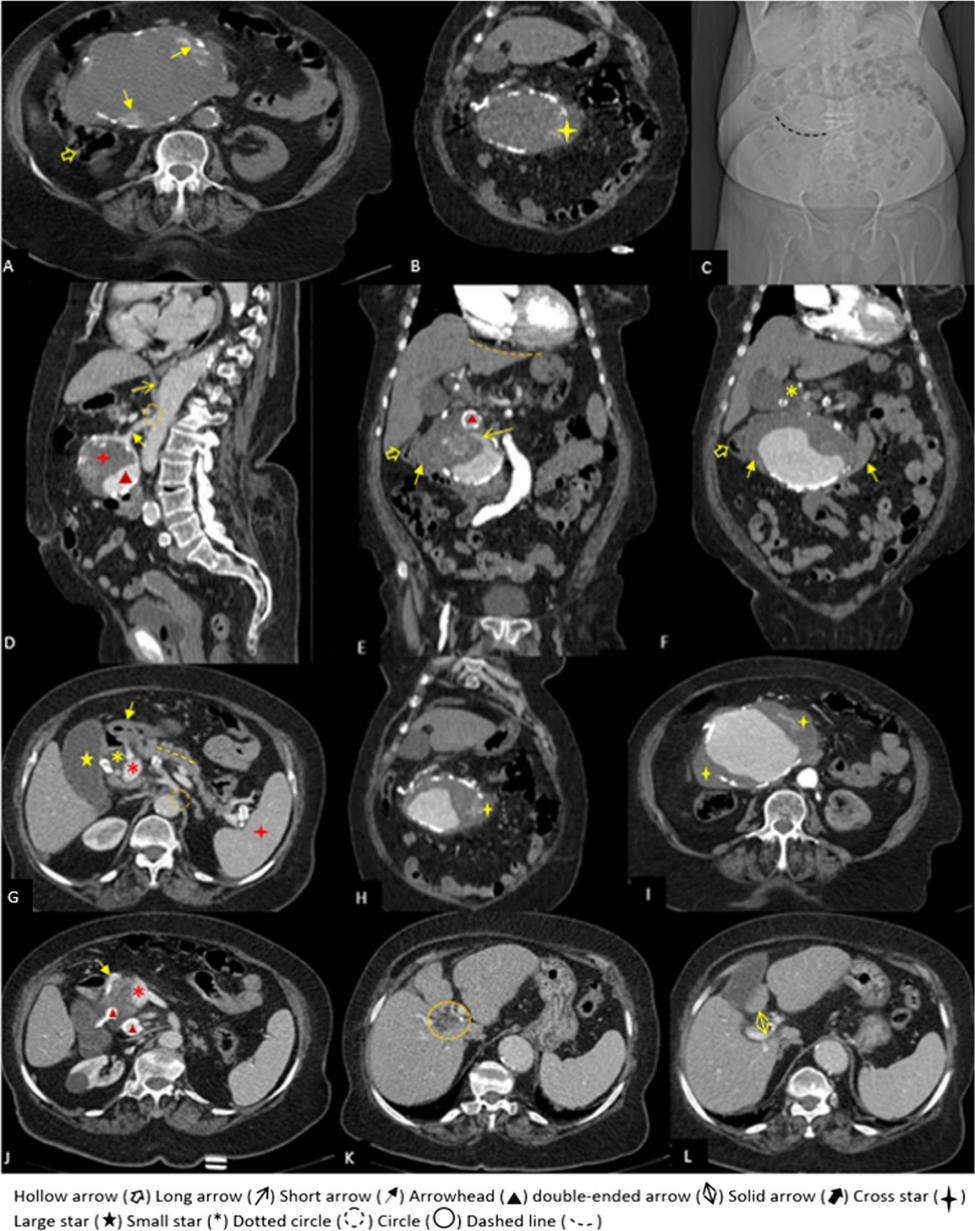
**A**, **B** non-contrast axial and coronal computed tomography scan show a huge hetero-dense mass in the sub-hepatic region, near to the head of the pancreas and medial to the cecum (yellow hollow arrow), with focal bulging at its medial margin (yellow cross star) and peripheral calcification and marginal hyper-dense crescent-like foci (short yellow arrows) suggestive of probably an aneurysm with hyperdense crescent sign. **C** Abdominal computed tomography scan Topogram: curvilinear calcification is visible mainly at right side of thoracolumbar spine in the sub-hepatic region. **D** Sagittal plane of computed tomography angiography, reveals an aneurysm with mural thrombosis (red cross star) and its patent lumen (red arrowhead) originating from the posterior and inferior surface of the proximal of the superior mesenteric artery (short arrow). The origin of the celiac trunk (dotted circle) is significantly narrowed due to the compressive effect of thickened diaphragmatic crura (long arrow). **E**, **F** Coronal plane of computed tomography angiography shows considerable compressive effect of huge aneurysm on the cecum, D2 and D3 portions of the duodenum (short yellow arrows) and common bile duct (small yellow star). A thrombus fissuration sign (long yellow arrow) is an extension of contrast from the patent lumen to the mural thrombus. The red arrowhead points to the another aneurysm, located at the superior margin of the giant aneurysm. The orange dashed line shows the slightly irregular liver’s margin. **G** Portal vein (small red star) and dilated common bile duct and are visible on the axial plane in the porta-hepatis region. The gallbladder is shown by the large star. The pylorus and D1 portion of the duodenum are indicated by the small yellow arrow. A yellow dashed line represents mild dilatation of the main pancreatic duct. The celiac trunk's origin has narrowed significantly (dotted circle). The red cross star represents the spleen. **H**, **I** The yellow cross stars indicate focal bulging and discontinuous calcification at the medial and lateral margins of the huge aneurysm. Mild fat stranding is present, adjacent to medial margin of the aneurysm. **J** The axial scan just inferior to "G". Gastroduodenal artery (GDA) is shown by short yellow arrow. Two aneurysms originating from the posterior branch of GDA (posterior superior pancreaticoduodenal artery) are depicted by the red arrowheads. (**K**, **L**) The central intrahepatic bile ducts are mildly dilated. The anterior periportal space is widened

**Fig. 3:**
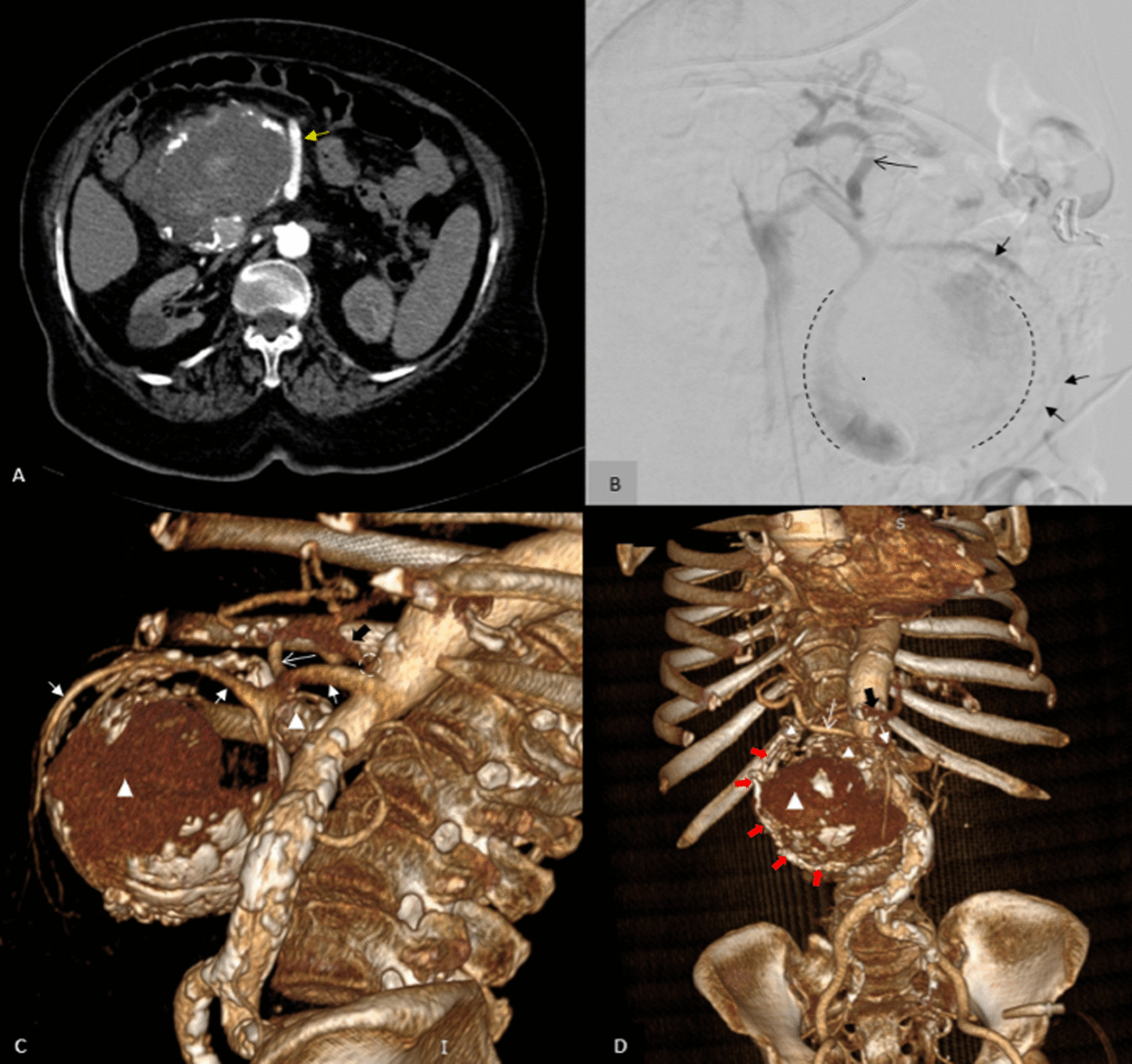
**A** The yellow arrow in the axial plane of computed tomography angiography indicates superior mesenteric artery, which continues its course to branches to supply small bowel. **B** Conventional angiography. Huge aneurysm originating from posterior and inferior surface of the proximal of superior mesenteric artery is seen in the lateral view of the superior mesenteric artery angiogram. The margin of this aneurysm is shown by the black dashed line. Short black arrows indicate the superior mesenteric artery itself, which is continuously branched in front of this aneurysm. A long black arrow indicates the right hepatic artery originating from superior mesenteric artery. **C**, **D** Reconstructed volume rendering images. White arrowheads point to three aneurysms, and short white arrows indicate superior mesenteric artery and its branches. Long white arrow indicates the right hepatic artery originating from superior mesenteric artery. Solid red arrows indicate anterior pancreaticoduodenal artery’s course which originates superiorly from gasrtoduodenal artery and is connected inferiorly to the huge aneurysm. A solid black arrow indicates the celiac trunk. The dotted circle shows obvous narrowing of the origin of the celiac trunk

In the non-contrast CT that was done before the computed tomography angiography (CTA), there was a huge mass (14 × 10 × 9 cm) with peripheral discontinuous calcification near the head of the pancreas extending superiorly to the subhepatic region and inferiorly medial to the cecum and causing severe compression on D2 and D3 portion of the duodenum and the common bile duct (CBD). Moreover, marginal hyperdense crescent areas were present (Fig. 2A–C). After contrast injection in the arterial phase of CTA, the mass had a density like an aorta and originated superiorly from the posterior surface of the proximal portion of the SMA. The aneurysm was inferiorly connected to the tortuous atherosclerotic vessels around the head of the pancreas. These vessels superiorly originated from the gastroduodenal artery. All arterial branches supplying the bowel loops originated from own SMA, not from this aneurysm. These findings suggest that the huge aneurysm originated from the inferior pancreaticoduodenal artery, which was connected to SMA on one side and to anterior and posterior pancreaticoduodenal arteries on the other (Figs. 2D, 3A, C, D).

There was mural thrombosis in the aneurysm, which was accompanied by a linear extension of intraluminal contrast through the mural thrombosis, indicating a thrombosis fissuration sign (Fig. 2E). Discontinuous calcification of the aneurysm's wall was present, and there were areas of focal bulging of the aneurysm's lumen to the surrounding mesenteric fat, associated with mild stranding and minimal fluid in these areas. Two smaller aneurysms (2.5 × 2.5 cm and 1.5 × 1.2 cm) were also seen adjacent to the superomedial margin of the huge aneurysm, originating from the posterior branch of the gastroduodenal artery (superior posterior pancreaticoduodenal artery) (Figs. 2E, J, 3C, D). A significant narrowing at the origin of the celiac trunk was noted due to diaphragmatic crura compression, indicating median arcuate ligament syndrome (Figs. 1D, 2D, G, 3C).

Although there was no obvious evidence of the aneurysm's rupture or extravasation of contrast but the presence of hyperdense crescent sign on non-contrast CT, thrombus fissuration sign on CTA, and areas of focal bulging of aneurysm's lumen associated with adjacent fat stranding were impending rupture signs of aneurysm which all of them necessitates the early treatment.

So, conventional angiography (CA) was done (Fig. 3) to evaluate the precise anatomy of aneurysms, confirm CTA findings, and to perform transarterial embolization (TAE), but CA was terminated early due to some reasons. First of all, it was impossible to interpret digital subtraction angiography (DSA) images due to blurring caused by the patient's coughing. More significantly, the patient experienced dizziness following a puncture of the femoral artery. When she became dizzy, her blood pressure was 90/60, and her heart rate was 100. Fortunately, with close observation, minimal hydration therapy (up to 200 cc of fluid), and leg elevation, her vital signs quickly returned to normal. Her hemodynamics had stabilized after three hours, and hemorrhagic shock was ruled out. Her symptoms were assumed to be the result of reflex syncope brought on by coughing and arterial puncture. After the patient's blood pressure and heart rate returned to normal, the angiographic procedure could be completed while she was under general anesthesia. However, we were leaning towards the surgical option due to the lack of patient and family consent for general anesthesia to perform supplementary angiography.

The massive size of the aneurysm (the largest diameter among previously reported cases) as well as the severe compression of the aneurysm on CBD causing chronic cholestasis leading to some degrees of chronic liver disease, were other reasons to do open surgery, which could theoretically resolve obstructive jaundice, and prevent the progression of chronic liver disease by resection of giant aneurysm and two other smaller aneurysms. Decompressing the celiac trunk from the diaphragmatic crura was also possible by the surgery plan.

Under general anesthesia, an anterior incision was made in the midline of the abdomen. The large aneurysm of the SMA branch had caused the elevation of the D3 and D4 parts of the duodenum. The duodenum was released, allowing access to the suprarenal and infrarenal aorta. The SMA artery was then exposed and released from the base of the transverse colon to a length of 5 cm. The distal portion of the SMA, beyond the replaced right hepatic branch (the first branch of the SMA), was explored and heparinized.

To preserve the blood supply to vital structures, such as the intestines, the anterior main branch of the SMA was meticulously preserved. However, the posterior branch of the SMA, from which the aneurysm sac originated, was clamped both proximally and distally to prevent further complications. Then, the aneurysm sac was carefully opened, resulting in the evacuation of multiple clots. In addition to the ligation of the proximal and distal ends of the aneurysm, the branches inside the aneurysm sac were also ligated to ensure complete hemostasis.

Following thorough washing and meticulous hemostasis, the huge aneurysm’s sac was covered. The same procedure was done for two smaller gastroduodenal arteries (GDA) branch aneurysms (posterior superior pancreaticoduodenal artery) after clamping of GDA (beyond the right gastroepiploic origin). Our concern about hepatic ischemia was resolved because of the presence of replaced right hepatic artery from SMA, which was preserved during surgery, and fortunately, no evidence of hepatic or biliary ischemia was seen after the surgery.

**Fig. 4:**
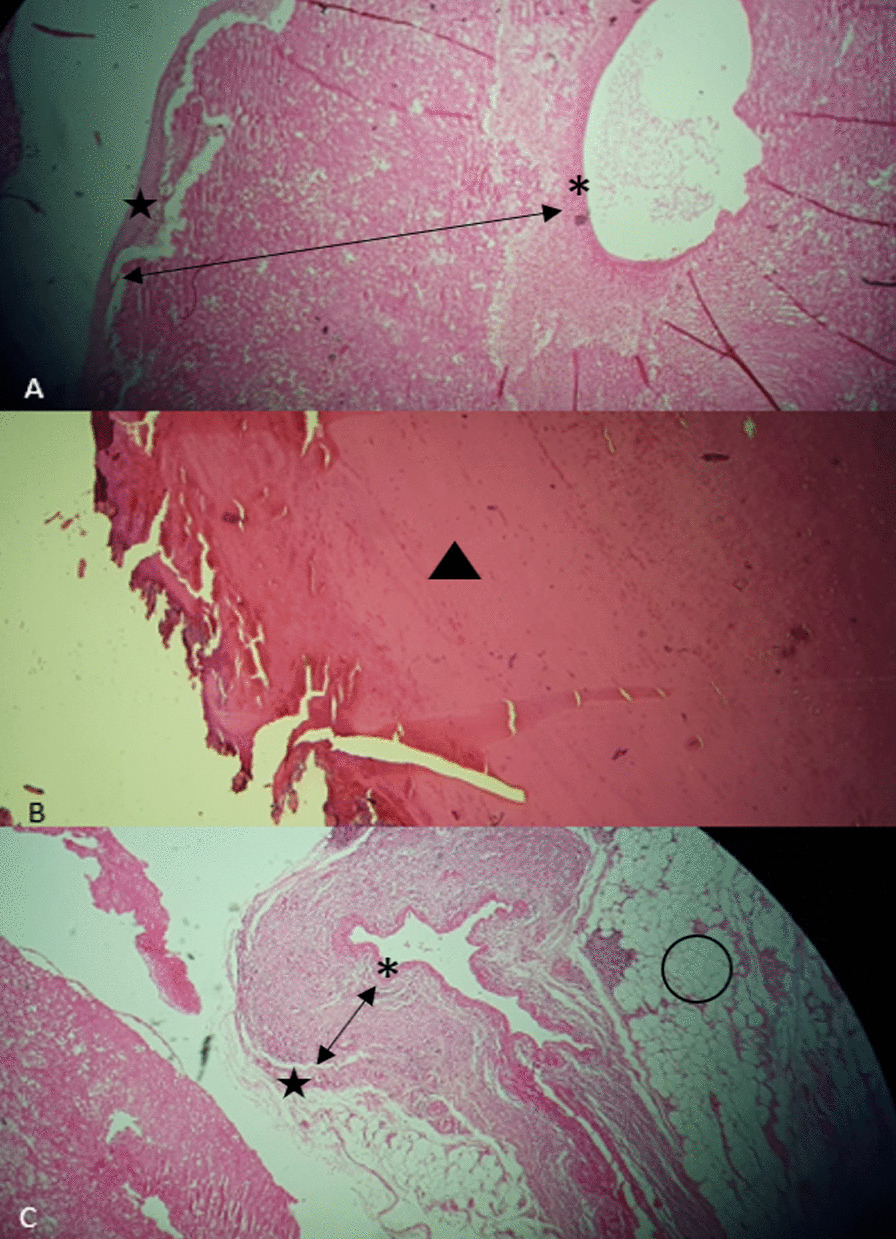
Some sections of the resected aneurysms are shown by H&E staining under the microscope. **A**, **C** The small star indicates the intima and the double-ended arrow indicates the media, which consists in part of hypertrophic smooth muscle and hyalinized necrosis. Large stars indicate an adventitial layer. The circle indicates adipose tissue. Fibrinoid necrosis and inflammatory cells were not seen in the resected aneurysms’ walls. **B** Black arrowhead indicates thrombus

Finally, transection of MAL and peri-arterial tissue was performed, and decompression of the celiac axis was done. After the division of MAL, blood flow was restored in the proper hepatic artery by palpation. In the end, the abdominal wall was repaired, and the patient was transferred to the recovery room and then surgery ICU. The next day after surgery, the patient suffered a sudden drop in blood pressure and shock and unfortunately passed away. Pathology of resected aneurysms confirmed the presence of all three layers of the arterial wall and the absence of inflammatory infiltrates, fibrinoid necrosis, or evidence of vasculitis in the arterial wall (Fig. 4). Unfortunately, the patient's family did not consent to an autopsy, but the most likely cause of death was hemorrhagic shock due to a blow out of a ligated artery in frail perivascular tissues.

## Discussion

The presented case described a rare cause of jaundice caused by the compressive effect of multiple pancreaticoduodenal aneurysms caused by median arcuate ligament syndrome. A few cases of obstructive jaundice caused by the compressive effect of PDAA have been reported to our knowledge (Table 1). 72.7% were male, and our case is the third known female patient with this condition. The mortality rate was 45.4%, and prior to our case, all deceased patients were male. The reported patients had an average age of 63 (between 48 and 84). Endovascular therapy was the most commonly used treatment method, either alone or in conjunction with surgery. Among the four previously reported deaths (as shown in Table 1), two were untreated, one was due to endovascular therapy, and one was due to surgical treatment.

**Table 1:** previous case reports of obstructive jaundice as a rare complication of pancreaticoduodenal artery aneurysm

No	Author	Year	Sex	Age	Presentation	CAS	Rupture	Mass	Diagnosis methods	Treatment	F/U
1	Sampsel *et al*. [15]	1952	M	68	Painless jaundice, pruritus, acholic stools, and dark-colored urine	NA	Yes	2.5 cm aneurysm of "anomaIous" artery ruptured into pancreas	Abdominal X-ray Laparatomy	Hepaticodochoenterostomy	D
2	Hasselgren *et al*. [16]	1976	F	62	Pain under the right costal margin, and jaundice	NA	No	Large aneurysm	Abdominal X-ray	Aneurysmectomy Exploratory choledochotomy	S
3	Scheflan *et al*. [17]	1977	M	56	Painless jaundice, pruritus, and malaise	Yes	Yes	Large hematoma and 2 cm aneurysm origin from the inferior PDA	Abdominal aortography Selective arteriography Upper GI series Ultrasonography	Aneurysmectomy Exploratory choledochotomy T-tube drainage	S
4	Kadir *et al*. [18]	1978	M	72	Gastrointestinal bleeding, jaundice, RUQ tenderness, and abdominal distention with ascites	Yes	No	2.5 × 3.5 cm aneurysm origin from inferior PDA	Selective arteriography	Untreated	D
5	Bécheur *et al*. [19]	1996	M	54	Jaundice, abdominal pain, and dilatation of the common bile duct	NA	NA	Peripancreatic pseudoaneurysm of the posterior and inferior PDA	Doppler ultrasonography Abdominopelvic CT scan	TAE	S
6	Widjaja *et al*. [20]	1999	M	51	Epigastric pain, jaundice, severe diarrhoea, and weight loss	NA	Yes	8.1 × 7.5 × 7.0 cm in left liver lobe as pseudoaneurysm of the PDA	Ultrasonography (B-mode and colour coded Duplex) Intraarterial digital subtraction angiography	PTBD TAE	S
7	Colak *et al*. [21]	2009	M	57	Persistent epigastric pain, weight loss, and jaundice	No	Yes	8.7 × 6.8 cm pseudoaneurysm origin from inferior PDA	Contrast-enhanced CT scan Non-selective abdominal angiography	Untreated	D
8	Wattez *et al*. [22]	2013	F	64	Intense acute abdominal pain, dilated intra- and extrahepatic biliary tracts (14 mm)	Yes	No	isolated 2-cm true aneurysm origin from PDA	Ultrasonography Contrast-enhanced CT scan	Laparotomy TAE (injection of polymeric synthetic into the aneurysm)	S
9	Yin *et al*. [11]	2014	M	84	RUQ pain, jaundice	Yes	Yes	Retroperitoneal hematoma and 2 cm aneurysm origin from PDA	Ultrasonography Abdominopelvic CT scan CT angiography Conventional angiography	TAE (with no need to drain the liver)	D
10	Otaegui *et al*. [23]	2016	M	48	Jaundice, epigastric pain, acholic stools, and dark-colored urine, dilated intra- and extrahepatic tracts	Yes	No	3.5 cm pseudoaneurysm origin from inferior PDA, which was anastomosed with the posterior PDA	Ultrasonography Abdominopelvic CT scan	TAE	S
11	Current study	2022	F	77	Jaundice, abdominal pain, and abdominal distention	Yes	No	14 × 10 × 9 cm true aneurysm, 2.5 × 2.5 cm true aneurysm, and 1.5 × 1.2 cm true aneurysm origin from posterior side of PDA	Ultrasonography Doppler ultrasound Abdominopelvic CT scan CT angiography Conventional angiography	Laparotomy	D

*PDAA* pancreaticoduodenal artery aneurysm, *PDA* pancreaticoduodenal artery, *RUQ* right upper quadrant, *PTBD* percutaneous transhepatic biliary drainage, *CT* computed tomographic, *TAE* transcatheter arterial embolization, *CAS* celiac axis stenosis, *M* male, *F* female, *F/U* follow up, *S* survival, *D* death, *GI* gastrointestinal

### Etiology and epidemiology

PDAA is the least common type of VAA, but has a high mortality rate and complications [24]. According to studies, the mortality rate in PDAA ranges from 26 to 50%. The most common cause of death in this condition is usually hemorrhagic shock caused by the rupture of an aneurysm (the risk is independent of size) [25, 26]. Etiologies of PDAA are classified into two broad categories based on whether the aneurysm is true or pseudo. A pseudoaneurysm is usually caused by abdominal trauma or pancreatitis, but a true aneurysm is usually due to celiac axis stenosis [11]. Arterial atherosclerosis and MALS are two potential causes of celiac axis stenosis, which leads to PDAA [27].

MALS, also known as Dunbar syndrome, is a condition in which the median arcuate ligament puts pressure on the celiac artery, where the abdominal aorta passes through the diaphragm, resulting in celiac artery obstruction or stenosis [28]. When the blood flow in the celiac trunk is decreased, the compensatory blood flow from SMA enters the PDA, leading to vascular hemodynamic disturbance. These hemodynamic changes can introduce excessive volume and pressure to the vessel wall and eventually lead to PDAA, which can be single or multiple [29, 30]. MALS is a relatively uncommon condition, affecting only 2 out of every 10,000 people [31]. This syndrome is mainly seen in thin women in their 4th or 5th decade of life, but the cause and other risk factors of this condition are still unclear [32, 33].

### Clinical manifestation

Although PDAA (as a type of VAA) cases are uncommon [10], clinical manifestations are more common in PDAA than in other types of VAA. If any clinical symptoms exist, they often include postprandial abdominal pain, nausea, vomiting, and shock due to ruptured aneurysms [24, 34]. Obstructive jaundice is reported as one of the atypical symptoms in some case reports. In Yin *et al*. study, eight cases were mentioned that suffered from jaundice due to the compressive effect of PDAA; half of these cases expired, and five cases had ruptured aneurysms. Obstructive jaundice can be caused by the compression effect of the PDAA itself or retroperitoneal and peripancreatic hematoma associated with ruptured PDAA [11]. If this condition becomes chronic, it can subsequently cause secondary biliary cirrhosis, as in the current report which some radiologic signs of chronic parenchymal liver disease were present. Because of this anatomical position, Schefflen *et al*. [17] considered PDAA as a rare differential diagnosis for obstructive jaundice in their presented case.

### Diagnosis

As an underlying cause of PDAA, MALS is usually diagnosed by Doppler ultrasound, CTA, and CA [35]. The first diagnostic modality is Doppler ultrasound, which can show accelerated velocities throughout expiration. Still, we could not do dynamic Doppler ultrasound due to inadequate patient cooperation. The complementary modality for diagnosing MALS is CTA or magnetic resonance angiography (MRA), which can show the compressive effect of the median arcuate ligament on the celiac artery [36]. This celiac artery narrowing usually looks like a hook and resembles the letter "J." On the other hand, an increase in the thickness of the median arcuate ligament of more than 4 mm is also abnormal and can be a sign of this syndrome [37]. CA is the gold standard diagnostic method for this syndrome [28].

PDAA is also usually found incidentally on imaging; most are readily diagnosed in cross-sectional imaging such as CT, CTA, or MRA [14]. Grey Scale and color Doppler ultrasound can be used to investigate an aneurysm's presence, especially for giant aneurysms. CTA is a suitable noninvasive diagnostic method for adequately identifying the relationship between aneurysms and related blood vessels, three-dimensional vascular reconstruction, and providing critical data for choosing the therapeutic method. CTA can also be used to assess evidence of PDAA rupture or signs of impending rupture [12]. A DSA is recommended in complex anatomical circumstances to evaluate an aneurysm and its arterial branch connections [38].

There are some CT-related features indicating that an aneurysm is an impending rupture. Siegel *et al*. performed a retrospective study on CT findings of ruptured and unruptured abdominal aortic aneurysms to determine if an aneurysm was at risk of rupture [39]. Larger anteroposterior and transverse dimensions, less thrombus, high-attenuation crescents within the mural thrombus, and focal discontinuity in otherwise circumferential calcification were all significant findings relating to impending rupture in this study.

Mehard *et al*. demonstrated the thrombosis fissuration in the wall of the aneurysm as a sign of impending rupture on CT/CTA. This finding is attributed to hemorrhage into thrombosis or aneurysm wall and blood leaking from the lumen into the thrombus, leading to the weakening of the aneurysm's wall. This sign indicates a forthcoming rupture, and immediate surgical intervention is mandatory. If blood transits the mural thrombosis and reaches the inner surface of the aneurysm's wall, it can perfuse the periphery of thrombosis along the intimal margin, producing a "hyperattenuating crescent" sign. This entity is associated with a 77% sensitivity and 93% specificity for rupture [40].

Focal bulging of the aneurysm's wall (bleb) also implies impending rupture and correlates to inflammatory changes and focal thinning of elastic fibers [41]. All of these impending rupture of aneurysm signs were present in our case.

### Treatment

Before delving into treatment options, it is critical to note that if PDAA is not treated, the mortality rate is 100%. So, regardless of the size of the PDAA, the patient should be treated [42]. To treat PDAA due to MALS, adequate blood flow to the organs supplied via the celiac axis should be preserved [43]. In general, there are two approaches to PDAA treatment: surgical procedures and endovascular therapy [44]. Surgical methods include ligation, resection, exclusion, and endoaneurysmorrhaphy, which were the preferred method in the past. Because the surgical method has a 50% mortality rate and a longer recovery period [45, 46]; on the other hand, the endovascular method is less invasive and has fewer complications [35]. Although endovascular methods are now the preferred method [47], Kubota *et al*. state that bypass surgery is preferable in unruptured PDAA caused by MALS if the stenosis is 80%, the length of the stenosis is 8 mm, or the distance between the stenosis and the aorta is less than 5 mm (which all of these indications were present in our case) [48].

TAE with coils is one of the most used interventional methods for the treatment of PDAA patients [49]. The main concerns regarding the use of TAE are the possibility of insufficient hepatic blood flow and aneurysm recurrence [50].

According to the previous case reports, a combination of surgical and endovascular methods can be used for some patients. This technique can be implemented in one step or two steps [43, 51]. For patients who require immediate hemostasis correction and adequate blood flow to upper abdominal organs such as the liver, a one-step method is used, in which emergency TAE is performed concurrently with bypass surgery. For patients with PDAA caused by MALS, the two-step method has been used in two ways: TAE followed by bypass surgery or bypass surgery followed by TAE [52].

The presence of multiple aneurysms added further complexity to this case. Due to the rarity of multiple PDAA, this condition's treatment is controversial. In a previous study, the surgical method was chosen in 50% of the cases with multiple aneurysms of the pancreatic arteries (7 out of 14 treated patients) [53].

Several methods have been used in previous case reports to treat obstructive jaundice caused by the external mechanical force of PDAA on the CBD. Yin *et al*. mentioned that endovascular therapy specific to PDAA usually resolves obstructive jaundice symptoms [11]. Otherwise, percutaneous transhepatic biliary drainage (PTBD) may be beneficial [54, 55]. Importantly, in obstructive jaundice caused by PDAA, endoscopic retrograde cholangiopancreatography (ERCP) carries a high risk of massive bleeding [56].

When deciding on a treatment method, special consideration should be given to each patient's unique condition as well as the precise location and origin of the aneurysm. All three therapeutic methods, including bypass surgery, TAE, and combined/hybrid methods, can be used [49]. The TAE or combined/hybrid methods seemed to be the best and also safe methods with a high success rate [43, 49]. Endovascular methods can also be used for large or multiple PDAA, as well as conditions that cause obstructive jaundice.

Although we decided to perform TAE first, unfortunately, this procedure could not be completed as planned. The large size of the largest aneurysm, evidence of severe compression effect of aneurysms on CBD leading to chronic parenchymal disease, the multiplicity of aneurysms, accompanying MALS, and the presence of surgery indications mentioned by Kubota *et al*. [48] were other factors that led us to choose a surgical plan for the next step. However, in similar cases, including giant or multiple PDAA cases with obstructive jaundice, we recommend using the endovascular or hybrid method under any circumstances.

## Conclusion

PDAA following celiac occlusion is one of the rarest causes of obstructive jaundice. So in patients with cholestatic jaundice accompanied by a mass in the pancreatic head with peripheral calcification, it is recommended not to forget this rare cause before invasive procedures such as ERCP, fine needle aspiration, and biopsy to prevent catastrophic hemorrhage. Due to the high mortality and morbidity associated with this condition, further evaluation with CTA is advised when this condition is suspected. The treatment method depends on each patient's clinical scenario and imaging findings and can be chosen between surgery or endovascular therapy, or a combined method.

## Data Availability

The datasets supporting the conclusions of this article is(are) included within the article and its Additional files.

## Appendix 1: Laboratory test report

**Table:**

Laboratory test	Result of laboratory test				
	Before diagnosis	One day before surgery	Immediately after surgery	One day after surgey	**Refrence value**
Hemoglobin	12	12.2	11.1	9.0	12–16
Aspartate aminotransferase (AST)	90	95	113	129	0–37
Alanine aminotransferase (ALT)	50	49	46	61	0–31
Alkaline phosphatas	1873	1650	703	394	64–306
Prothrombin time (PT)	14	17.7	18.9	17	11–13.9
Partial Thromboplastin Time (PTT)	76	70	98	91	25–45
international normalised ratio (INR)	1.2	1.29	1.38	1.24	0.9–1
Bilirubin (total)	30	18.3	14.8	12.5	0.2–1.2
Bilirubin (direct)	16	10.3	9.2	8.5	0–0.4
Lactate dehydrogenase (LDH)	434	449	422	450	Up to 480
Amylase	12	43	23.7	51	Up to 100
Albumin	2.7	2.5	2.6	2.6	3.5–5.2

## Abbreviations

ALT: Alanine aminotransferase
AST: Aspartate aminotransferase
CA: Conventional angiography
CBC: Complete blood count
CBD: Common bile duct
CRP: C-reactive protein
CT: Computed tomography
CTA: Computed tomography angiography
DSA: Digital subtraction angiography
ERCP: Endoscopic retrograde cholangiopancreatography
ESR: Erythrocyte sedimentation rate
ICU: Intensive care unit
MALS: Median arcuate ligament syndrome
MRA: Magnetic resonance angiography
PDA: Pancreaticoduodenal artery
PDAA: Pancreaticoduodenal artery aneurysm
PTBD: Percutaneous transhepatic biliary drainage
SMA: Superior mesenteric artery
TAE: Transarterial embolization
VAA: Visceral artery aneurysm
